# Physicochemical Properties Analysis and Secretome of *Aspergillus niger* in Fermented Rapeseed Meal

**DOI:** 10.1371/journal.pone.0153230

**Published:** 2016-04-06

**Authors:** Changyou Shi, Jun He, Jie Yu, Bing Yu, Xiangbing Mao, Ping Zheng, Zhiqing Huang, Daiwen Chen

**Affiliations:** Animal Nutrition Institute, and Animal Disease-Resistance Nutrition Key Laboratory of Sichuan Province, Sichuan Agricultural University, Ya’an 625014, China; Korea University, REPUBLIC OF KOREA

## Abstract

The nutrient digestibility and feeding value of rapeseed meal (RSM) for non-ruminant animals is poor due to the presence of anti-nutritional substances such as glucosinolate, phytic acid, crude fiber etc. In the present study, a solid state fermentation (SSF) using *Aspergillus niger* was carried out with the purpose of improving the nutritional quality of RSM. The chemical composition and physicochemical properties of RSM before and after fermentation were compared. To further understand possible mechanism of solid state fermentation, the composition of extracellular enzymes secreted by *Aspergillus niger* during fermentation was analysed using two-dimentional difference gel electrophoresis (2D-DIGE) combined with matrix assisted laser desorption ionization—time of flight—mass spectrometer (MALDI-TOF-MS). Results of the present study indicated that SSF had significant effects on chemical composition of RSM. The fermented rapeseed meal (FRSM) contained more crude protein (CP) and amino acid (AA) (except His) than unfermented RSM. Notably, the small peptide in FRSM was 2.26 time larger than that in unfermented RSM. Concentrations of anti-nutritional substrates in FRSM including neutral detergent fiber (NDF), glucosinolates, isothiocyanate, oxazolidithione, and phytic acid declined (*P* < 0.05) by 13.47, 43.07, 55.64, 44.68 and 86.09%, respectively, compared with unfermented RSM. *A*. *niger* fermentation disrupted the surface structure, changed macromolecular organic compounds, and reduced the protein molecular weights of RSM substrate. Total proteins of raw RSM and FRSM were separated and 51 protein spots were selected for mass spectrometry according to 2D-DIGE map. In identified proteins, there were 15 extracellular hydrolases secreted by *A*. *niger* including glucoamylase, acid protease, beta-glucanase, arabinofuranosidase, xylanase, and phytase. Some antioxidant related enzymes also were identified. These findings suggested that *A*. *niger* is able to secrete many extracellular degradation enzymes (especially lignocellulosic hydrolyzing enzymes, acid proteases and phytase) during fermentation of RSM, thus altering chemical composition and physicochemical properties of RSM.

## Introduction

Rapeseed is the world’s third largest source of vegetable oil. Rapeseed meal (RSM) is the by-product of oil extraction from seeds and is mainly composed of proteins, lignocellulosic fiber and minerals [1]. In China, the production of RSM was estimated at about 11.4 million tons in 2014. RSM is a good protein resource for animal feed, the amino acid balance of RSM is the best of the commercial vegetable protein sources currently available [2]. However, the nutrient digestibility and feeding value of RSM for non-ruminant animals is poor due to the presence of anti-nutritional substances such as glucosinolate, phytic acid, crude fiber etc. [3]. Glucosinolates are hydrolyzed by myrosinase enzyme present in the rapeseed or animal gastrointestinal tract to release a range of products including isothiocyanate, thiocyanate and nitrile. These degradation products impair palatability, affect liver and kidney functions, and interfere with iodine availability [4]. Phytate may reduce mineral bioavailability and might reduce protein digestibility [5]. What more, RSM contains relatively high levels of fiber which may accelerate the digesta passage rate and result in reduced time for digestion and thus reduced nutrient utilization [3].

Solid state fermentation (SSF) is defined as the fermentation involving solids in near absence of free water; however, substrate must possess enough moisture to support growth and metabolism of microorganism. Solid state fermentation (SSF) has emerged as a potential technology for utilisation of agro-industrial residues. At the beginning of the 1994s, SSF was employed for detoxification of RSM. Bau et al (1994) found that SSF with *Rhizopus oligosporus* for 24 h resulted in the degradation of 57.7% aliphatic glucosinolates, 97.3% indol glucosinolates and 73% ethanol-soluble sugars of RSM [6]. Smit et al (1994) developed a fuzzy model to predict the glucosinolate content of RSM during SSF [7]. Solid state fermentation (SSF) has also been reported to be an effective way to reduce phytic acid and fiber of RSM [8–9]. *Aspergillus niger*, generally recognised as a safe (GRAS) microorganism, posses the capacity to synthesize proteases, amylases, fibre degrading enzymes (cellulases, hemicellulases, pectinases), lipases, and tannase [10]. In 2013, *Aspergillus niger* has been allowed to add in feed for animal production by the ministry of agriculture of the people’s republic of China. Recent studies found that SSF with *A*. *niger* could degrade anti-nutritional substances of rapeseed cake/meal and cassava meal and upgrade nutritional value of substrate [2, 11–12]. However, mechanism of SSF is still not clear and needs to be further studied. To further understand the effects of solid state fermentation on physicochemical properties of RSM, microstructure of rapeseed meal before and after fermentation was observed by scanning electron microscope (SEM). Fourier transform infrared (FTIR) spectroscopy was performed in order to explore the fate of organic matters during the fermentation. Protein molecular weight change during fermentation was evaluated by SDS-PAGE. The composition of extracellular enzymes secreted by *A*. *niger* during fermentation was analyzed using two-dimensional difference gel electrophoresis (2D-DIGE).

## Materials and Methods

### Microorganism, basal substrate and Solid state fermentation

*A*. *niger* (CICC 41258) was obtained from China Center of Industrial Culture Collection (CICC) and maintained on potato dextrose agar (PDA) slants at 4°C. *A*. *niger* spores were washed from 5–7 days agar slants with 0.2% Tween 80. The concentration of spores was counted by using a counting chamber.

Rapeseed meal (RSM) and wheat bran used in the study were obtained from Xinxin Grains & Oils (Group) Co. Ltd., Chengdu, China. The RSM and wheat bran were dried in an oven at 105°C to constant weight. Then, they were ground to pass a 40-mesh sieve.

**Fig 1:**
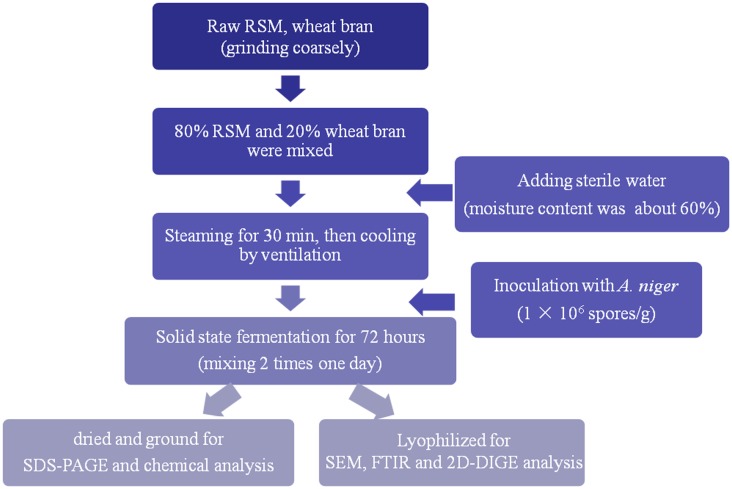
Schematic outline of the manufacturing process of FRSM.

Fig 1 shows the schematic outline of the manufacturing process of fermented rapeseed meal (FRSM). The quanity of substrate was 100 kg for dry basis. The basal substrates including 80% RSM and 20% wheat bran were mixed and inoculated with spore suspension (1 × 10^6^ spores/g) of *A*. *niger*, fermented in a bed-packed incubator at 34°C for 72 h. The initial moisture content was 60% in dry basis. After fermentation, typical samples were collected from four different parts of the piles, then some of which was lyophilized and stored at 4°C for SEM, FTIR and 2D-DIGE analysis, and the rest of fermented substrate was dried at 105°C for 30 min to to collapse the porosity of biomass needed to the fungal growing and development, then was dried at 55°C for 48 h, cooled and ground to pass a 60-mesh sieve for SDS-PAGE and chemical analysis. The control of RSM was autoclaved too in our study in order to separate the effect of fungal fermentation on glucosinolates.

### Chemical analysis

The FRSM and untreated RSM samples were analyzed for dry matter (DM), crude protein (CP), ether extract, neutral detergent fiber (NDF) [13], acid detergent fiber (ADF), ash, calcium (Ca) and phosphorus (P) content according to AOAC 2005 [14]. The amino acid (AA) profiles of samples were analyzed using AA analyzer (Hitachi L8800, Tokyo, Japan). Before analysis, samples were hydrolyzed with 6 mol/L HCl for 24 h at 110°C, Methionine and Cys were analyzed as Met sulfone and cysteic acid after cold performic acid oxidation overnight before hydrolysis.

Small peptide was calculated by subtracting free amino acid (FAA) from trichloroacetic acid—soluble protein (TCA-SP) [15]. The TCA-SP of samples were determined by Ovissipour et al. 2009 [16]. Three gram of sample was mixed with 25 mL 15% TCA, gently stirred for 20 min at room temperature and centrifuged at 4000 × *g* for 15 min. Subsequently, the supernatant was collected, the nitrogen content of the supernatant are then determined by the Kjeldahl method using auto-Kjeldahl's apparatus (FOSS 2200, Foss, Denmark). The FAA in FRSM or RSM were extracted with 0.02 mol/L of HCl and determined by amino acid (AA) Analyzer (model L8800, Hitachi).

The glucosinolates content of samples were determined according to palladium chloride method [17]. The 2 mL of RSM or FRSM extract solution was added to a tube containing 6 mL of 0.1% sodium carboxymethylcellulose and 1.4 mM palladium chloride. The absorbance was determined using a spectrophotometer (7200, UNICO, China) at 540 nm (E1) by using sodium carboxymethyl cellulose and palladium chloride as a reference solution. Another 2 mL of RSM or FRSM extract was added to a test tube containing 6 ml of 0.1% sodium carboxymethylcellulose. The absorbance at 540 nm (E2) was measured by using sodium carboxymethylcellulose and distilled water as a reference solution. Glucosinolates content was assessed by the absorbance value E (E = E1 − E2), which is proportionate to the content the latter being measured by a sinigrin standard curve. Total isothiocyanates was determined by UV spectrophotometry (Cecil, CE 7200 England) according the mehod developed by Choi et al. 2004 [18]. Phytate in samples analyzed by ferric chloride colorimetric menthod as described by Ellis et al. 1977 [19].

### Microscopic observation

Scanning electron microscopy (SEM) was used for study of the effect of pretreatments on physical property changes in the biomass. Microstructure of raw RSM and FRSM was observed using a field-emission scanning electron microscopy (JSM-7500F, JEOL, Japan) at 400× and 3000× magnification. The freeze-dried samples were placed on an aluminium stub and coated with gold. The micrographs were taken using an Everhart-Thornley (E-T) detector at 15 kV and a high vacuum mode.

### Fourier transform infrared (FTIR)

The structural properties of raw and fermented RSM were analyzed by FTIR spectrometer (Nicolet 6700, Thermo Scientific, USA). Sample discs were prepared by mixing 2 mg dried samples with 200 mg KBr and then pressing the mixture at 10 MPa for 5min. The FTIR Spectra in the range of 400–4000 cm^−1^ were obtained by averaging 16 scans at a resolution of 4 cm^−1^. In the present study, two regions (3400–3000 cm^-1^, and 1800–900 cm^-1^) which represent main bands of cellulose, protein and polysaccharide were focused upon according to previous report [20].

### Sodium dodecyl sulfate–polyacrylamide gel electrophoresis (SDS-PAGE)

Soluble proteins in FRSM and unfermented RSM were extracted according to Faurobert [21] with minor modification. The samples were ground finely to pass a 60-mesh sieve. 1.5 mL of 20 mM Tris-HCl buffer (pH 7.6) including 5 μg/mL protease inhibitor, 0.1% SDS and 5 mM dithiothreitol was added to each 100 mg of the ground samples, then homogenized on ice with for 10 minutes. The homogenized samples were centrifuged at 11,000 × g for 20 minutes at 4°C (5804R, Eppendorf, Germany), then supernatants solutions were transferred to 1.5 mL Eppendorf (EP) tubes. Protein concentration in each sample was determined using Bio-Rad Protein Assay Kit (Bio-Rad, USA) according to the manufacturer's instructions. Soluble protein was fractionated by SDS-PAGE system according the previous method [22]. The electrophoresis system was based on 12% polyacrylamide separating gels containing 0.1% SDS in Tris-glycine buffer. About 20 μg of extracted protein sample was loaded for each well and separated at 65 mV for 120 minutes. After electrophoresis, the gel was stained using Coomassie Brilliant Blue (CBB) R-250 (Bio-Rad, USA) for 45 minutes and de-stained with 7% acetic acid.

### Preparation of the secretome for 2D-DIGE

The extracellular protein of *A*. *niger* was prepared according the method described by Zhang et al. 2012 [23]. 25 g FRSM was soaked overnight in 50 mL of acetate buffer (50 mM, pH 5.0) containing 5 μg/mL protease inhibitor (Roche, Switzerland) at 4°C. The suspension was centrifuged at 11,000 × g for 15 min and filtered through a 0.45 μm filter (Millipore, USA). The soluble protein samples were concentrated by ultrafiltration (Cut-off 10 kDa, Millipore, USA). 10 μL of 20 mg/mL sodium deoxycholate were added in 1 mL prepared protein solution, vortexed and kept for 30 min on ice. Then, TCA was added to a final concentration of 12% (w/v) and the mixture was incubated for 1 h at 4°C. The precipitation was collected at 15,000 × g for 1 h at 4°C. The protein pellet was washed twice with cold acetone to remove TCA, centrifuged, and solubilized in lysis buffer containing 7 M urea, 4% (w/v) CHAPS, 1% (w/v) DTT, and 1% (v/v) IPG buffer pH 4–7 (GE Healthcare, USA). The negative control was unfermented RSM, upon which 2-DE was also performed under the same conditions. The protein concentration was determined using the BIORAD protein assay (BIO-RAD, USA) according to the manufacturer's protocols. The solubilized proteins were stored at -80°C until further analysis by 2D-DIGE.

### Two-dimensional gel electrophoresis

Isoelectric focusing (IEF) was run with the IPGphor™ 3 Isoelectric Focusing System (GE Health, USA) at 20°C with 45 μA per strip. The protein sample (300 μg) was loaded onto immobilized pH gradient (IPG) strips gels of pH 4–7 (GE Healthcare, USA). The IPG strips were focused at gradient steps of 50 V × 12 h, 500 V × 1 h, 1000 V × 1 h, gradient from 1000 V to 5000 V within 2 h, 5000 V × 2 h, gradient from 5000 V to 10000 V within 2 h, then 10000 V × 12 h. Before second-dimension separation, the IPG strips were incubated in equilibrated buffer containing 50 mM Tris-HCl (pH 8.8), 6 M urea, 2% (w/v) SDS, 1% DTT (w/v) and 30% (v/v) glycerol for 15 min. Then, the strips were equilibrated in the same equilibrated buffer additionally containing 2.5% (w/v) iodoacetamide for 15 min. The IPG strips were subsequently transferred to the top of lab cast SDS-polyacrylamide gels. The SDS-PAGE was then carried out using the Ettan DALT 6 electrophoresis system (GE Healthcare, USA) in constant working voltage as follows: 100 V for 45 min and 200 V for 8 h until bromophenol blue (BPB) band reached bottom of the gels. Subsequently, the gels were fixed for 2 h and stained overnight with CBB—G250 solution (0.1% CBB—G250, 10% phosphoric acid, 20% methanol, and 10% ammonium sulfate). The stained gels were scanned using an Image Scanner (GE Healthcare, USA) at a resolution of 300 dots per inch. All gel images were processed by three steps: spot detection, quantification, and matching, using PDQuest 8.0 software (Bio-Rad, USA).

### Protein identification by MALDI-TOF/TOF tandem MS

Compared with the 2D maps of unfermented RSM (negative control), the protein spots only observed in FRSM 2D maps were excised from the gels and destained with 25 mM NH_4_HCO_3_ in 50% acetonitrile (ACN) for 30 min. After being washed twice with 100% ACN for 10 min, the proteins were digested in-gel using 0.02 μg/μL trypsin (Promega, Madison, USA) in 25 mM NH_4_HCO_3_ and 10% ACN overnight at 37°C. Then, digested samples were extracted with 5% TFA and 67% ACN at 37°C for 30 min, 5,000 × g for 5 min. The peptide extracts and the supernatant of the gel spot were combined and completely dried. The samples were re-suspended with 5 μL 0.1% TFA and spotted on a stainless steel target plate. Peptide MS and MS/MS analyses were performed using an ABI 5800 MALDI-TOF/TOF Plus mass spectrometer (Applied Biosystems, USA). Data were integrated and processed by using GPS Explorer V3.6 software (Applied Biosystems, USA). The proteins were successfully identified with a 95% or higher confidence interval using the MASCOT V2.3 search engine (Matrix Science, UK) and searching in the annotated *Aspergillus niger* genome (EMBL: http://www.ebi.ac.uk/genomes/eukaryota.html) serving as database [24]. The search parameters were set as follows: trypsin as the digestion enzyme; one missed cleavage site; fixed modifications of carbamidomethyl; partial modifications of acetyl, deamidated, dioxidation, oxidation; peptide tolerance at 100 ppm and fragment mass tolerance of 0.3 Da. Mowse score greater than 53 was regarded as significant (*P* < 0.05). The annotations were in accordance with the genome of *A*. *niger* and NCBI Reference Sequence database.

### Statistical analysis

Data obtained from chemical analysis of FRSM and unfermented RSM samples were analyzed by one-way analysis of variance (ANOVA) following the General Linear Models (GLM) procedure of the SAS software (SAS, 1999). Differences between two means were tested using Student’s T-test. A significant level of 0.05 was used as indication of a difference.

## Results

### Chemical Composition

Analyzed nutrient contents of the unfermented RSM and FRSM are presented in Table 1 (S1 Table for raw data in Table 1). The FRSM contained more CP, ash, Ca, total P, and AA (except His) than unfermented RSM. However, the crude fat was lower for FRSM than that for unfermented RSM. The content of small peptides in unfermented RSM was 2.57% respectively, whereas in FRSM, that content was 8.39%. Concentrations of NDF, glucosinolates, isothiocyanate, oxazolidithione, and phytic acid in FRSM declined (*P* < 0.05) by 13.47, 43.07, 55.64, 44.68 and 86.09%, respectively, compared with unfermented RSM.

**Table 1:** Analyzed nutrient composition of fermented rapeseed meal (FRSM) and unfermented rapeseed meal (RSM), as DM basis^1^.

Item	RSM	FRSM
CP, %	34.84 ± 1.93^b^	40.72 ± 2.71^a^
Small peptide^2^, %	2.57 ± 0.37^b^	8.39 ± 1.56 ^a^
Fat, %	4.11 ± 0.45	3.58 ± 0.40
Starch,%	3.71 ± 0.40^a^	1.22 ± 0.23^b^
NDF, %	47.08 ± 3.00 ^a^	40.74 ± 1.73 ^b^
ADF, %	33.70 ± 3.25	30.76 ± 3.17
Hemicellulose,%	13.39 ± 0.26 ^a^	9.98 ± 1.79^b^
Lignin,%	16.98 ± 2.14	15.12 ± 2.40
Ash, %	8.03 ± 0.44	9.30 ± 0.76
Ca, %	0.62 ± 0.08	0.76 ± 0.13
Total P, %	1.10 ± 0.16	1.35 ± 0.11
Glucosinolates, μmol/g	41.91 ± 5.17^a^	23.86 ± 4.25^b^
Isothiocyanate, mg/g	2.48 ± 0.46 ^a^	1.10 ± 0.19 ^b^
Oxazolidithione, mg/g	1.88 ± 0.23 ^a^	1.04 ± 0.16 ^b^
Phytic acid,%	2.66 ± 0.39 ^a^	0.37 ± 0.07 ^b^
Indispensable AA, %		
Arg	1.96 ± 0.32	2.06 ± 0.39
His	0.85 ± 0.14	0.78 ± 0.08
Ile	1.15 ± 0.08^b^	1.43 ± 0.13^a^
Leu	2.21 ± 0.15^b^	2.72 ± 0.25^a^
Lys	1.74 ± 0.34	1.86 ± 0.22
Met	0.52 ± 0.05	0.66 ± 0.13
Phe	1.31 ± 0.08 ^b^	1.65 ± 0.19^a^
Thr	1.38 ± 0.09 ^b^	1.69 ± 0.15 ^a^
Val	1.52 ± 0.26	1.76 ± 0.28
Dispensable AA, %		
Ala	1.31 ± 0.11^b^	1.73 ± 0.23^a^
Asp	2.17 ± 0.25^b^	3.04 ± 0.24 ^a^
Cys	0.66 ± 0.12	0.75 ± 0.14
Glu	6.16 ± 1.24	6.79 ± 1.08
Gly	1.56 ± 0.14^b^	1.91 ± 0.15^a^
Pro	1.89 ± 0.21^b^	2.25 ± 0.06^a^
Ser	1.43 ± 0.09	1.73 ± 0.19
Tyr	1.09 ± 0.09^b^	1.33 ± 0.08 ^a^

^1^ Values are means of three replicates per treatment. Means in a row without common superscript differ significantly (*P* < 0.05).

^2^ Small peptide = TCA-SP–free AA.

### Microscopic observation

**Fig 2:**
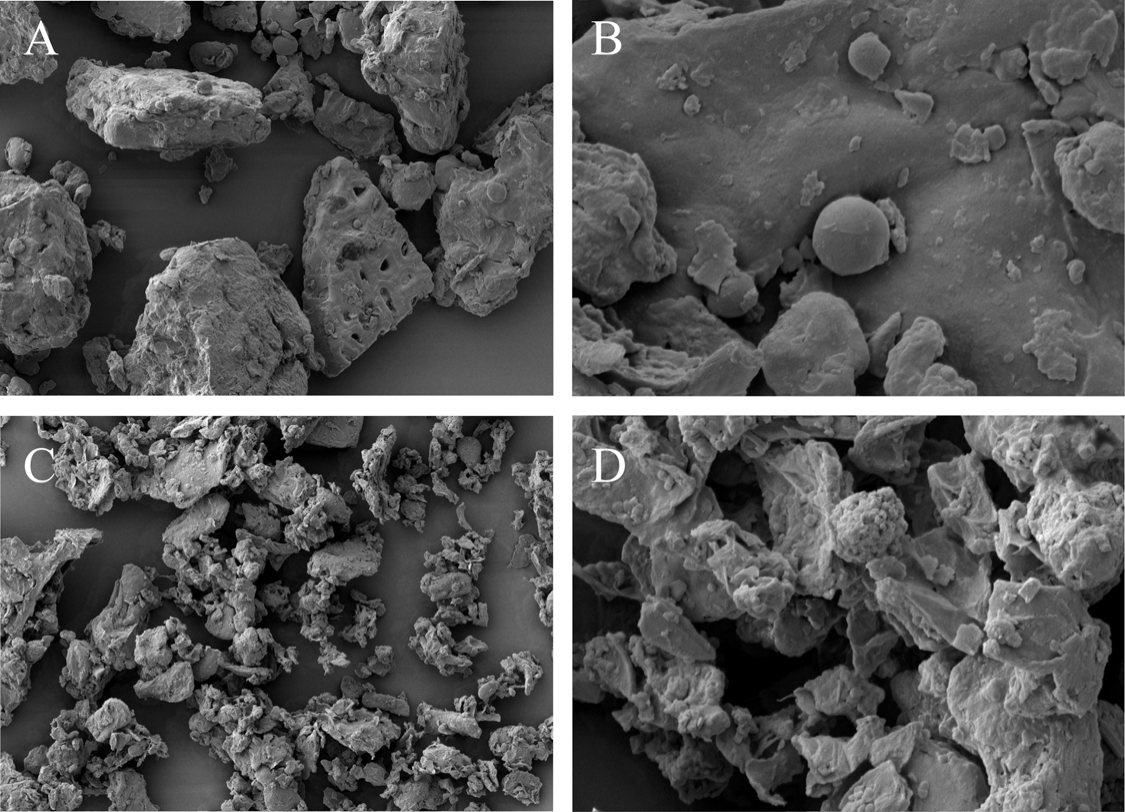
SEM micrographs of unfermented RSM and FRSM. A and B: unfermented rapeseed meal (rapeseed meal 80%, wheat bran 20%); C and D: fermented rapeseed meal; Magnifications of photographs are 400 × (A and C), and 3000 × (B and D).

Microstructure was clearly different between FRSM and untreated RSM (Fig 2). Irregular shape and rough surface were mainly observed in FRSM whereas microstructure of untreated RSM was regular and surface was smooth. Small amounts of starch granules was observed in raw RSM, after 3 d fermentation, starch granules was disappeared in FRSM.

### FTIR spectra

**Fig 3:**
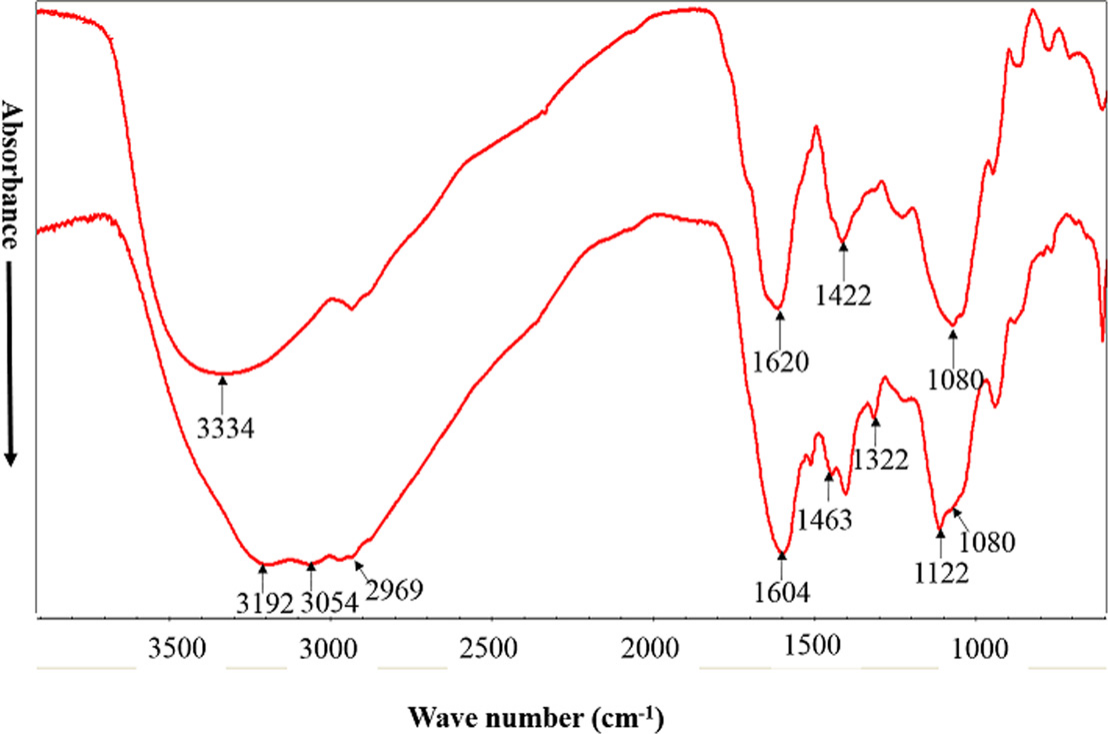
FTIR spectra of unfermented RSM and FRSM. A: unfermented rapeseed meal (rapeseed meal 80%, wheat bran 20%); B: fermented rapeseed meal.

For untreated RSM, the FTIR spectrum (Fig 3) presented peaks at 3334 cm^-1^ (OH), 1620 cm^-1^ (CO, CN), 1422 cm^-1^ (CH2) and 1080 cm^-1^ (OH), which indicate the presence of cellulose (3334 cm^-1^ and 1422 cm^-1^), proteins (1620 cm^-1^) and polysaccharides (1422 cm^-1^ and 1080 cm^-1^). In FTIR spectra of FRSM, several new absorbance at about 3192, 3054, 2969, 1463, 1322 and 1122 cm^−1^ were observed. In addition, the absorption peaks at 1604 cm^−1^, which is related to C = O stretching of amide groups in proteins or protein-like compounds, was increased after solid state fermentation. Compared to FTIR spectra of raw RSM, the absorbance at 1080 cm^−1^, which represents C-O stretching of polysaccharides or hemicelluloses substances, decrease after fermentation with *A*. *niger*.

### SDS-PAGE

**Fig 4:**
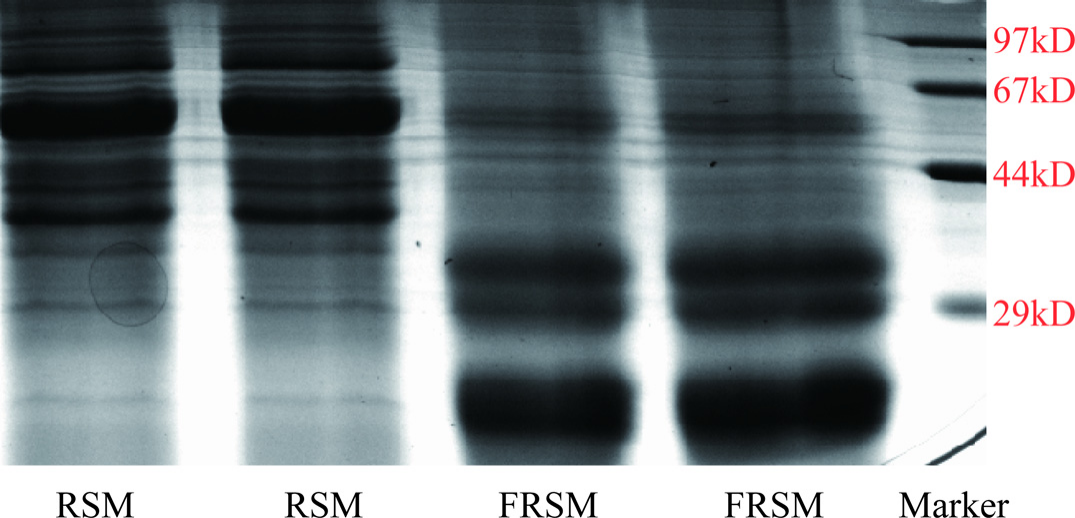
Distribution of peptides in rapeseed meal (RSM) before and after fermentation by A. *niger*. Lane M: protein MW markers (20–97 kDa); Lane 1 and 2: untreated rapeseed meal (rapeseed meal 80%, wheat bran 20%); Lane 3 and 4: fermented rapeseed meal.

Solid state fermentation with *A*. *niger* affected the characteristics of proteins in RSM (Fig 4). For unfermented RSM, rapeseed protein correspond to multiple bands in the range of 29–97 kDa. The molecular weight of main protein fractions in the unfermented RSM were 72, 55 and 37 kDa. Fermentation increased the amount of small-size peptides (< 35 kDa) compared with untreated RSM, while significantly decreasing large-size peptides (> 60 kDa).

### Extracellular protein analysis during solid state fermentation with *Aspergillus niger*

**Fig 5:**
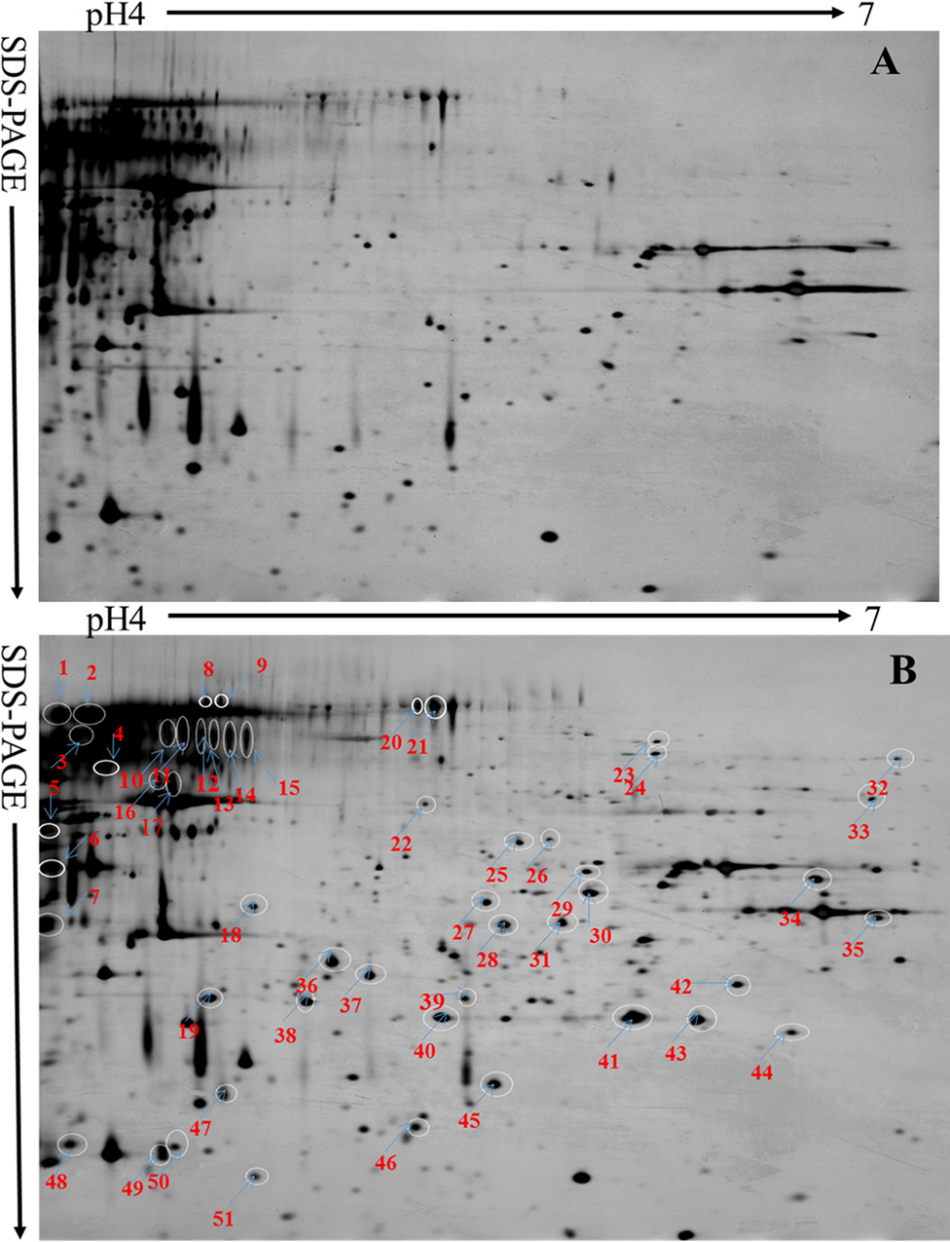
CBB-stained 2-DE maps. A: The 2-DE map of the negative control (the unfermented rapeseed meal); B: The 2-DE map of the secretome of *A*. *niger* CICC41258; The identified protein spots were numbered in order. Three independent experiments were performed.

The secretome of A. *niger* CICC 41258 in FRSM was separated using 2D-DIGE (Fig 5B). The analysis of the negative control (raw RSM) showed that there were fewer protein spots from unfermented RSM in the 2-DE maps (Fig 5A) than that from fermented RSM. A total of 51 protein spots on the 2-DE gel were excised, digested with trypsin, and analyzed by MALDI-TOF/TOF tandem MS. Among them, 40 (78.4%) were successfully identified. The searches of the identified protein spots with the annotated *A*. *niger* genome database showed that they represented 29 unique proteins (Table 2). Many unique proteins were well-known hydrolytic enzymes that are involved in the degradation of starch, protein, lignocellulose, etc. There were four spots (2, 8, 9 and 17) that belonged to glucoamylase (amyA) and their proteolytic products in the secretome of *A*. *niger* CICC 41258 in FRSM. Three aspartyl protein-degrading enzymes (spots 5, 6 and 7) with different abundances, including Aspergillopepsin A (Pep A) and Aspergillopepsin B (Pep B), were found in the secretome. Various cell wall-degrading enzymes were identified in the secretome including Endo-1,4-beta-xylanaseB (XynB), Alpha-L-arabinofuranosidase (AbfA), and Endo-1,3(4)-beta-glucanase (Eng). Other well-known extracellular proteins found in the secretome were Phytase (PhyA) and Carboxylesterase (Ces). Apart from the above described extracellular proteins, 17 intracellular proteins were also identified in the *A*. *niger* secretome in the present study.

**Table 2:** Composition of extracellular hydrolase and other related protein secreted by *Aspergillus niger* during solid state fermentation^1^.

Spots number	protein	Molecular weight (kDa)	PI^2^	MASCOT score^3^
extracellular hydrolase				
1	Alpha-L-arabinofuranosidase (AbfA)	68.02	4.10	109
2	Glucoamylase G1 (Gla-G1)	65.96	4.19	169
3/4	Beta-1,3-glucanosyltransferase (Gel3)	57.96	4.40	119/66
5/6	Aspergillopepsin A (PepA)	41.37	4.43	339/775
7	Aspergillopepsin B (PepB)	34.28	4.04	376
8/9	Glucoamylase A (GlaA)	68.91	4.28	314/212
11	Carboxylesterase (Ces)	58.17	4.53	149
12/13	Phytase (PhyA)	56.28	4.69	153/102
15	Endo-1,3(4)-beta-glucanase (Eng)	50.71	4.83	225
17	Alpha-Amylase (AmyA)	52.97	4.48	216
49	Endo-1,4-beta-xylanaseB (XynB)	22.64	4.31	106
Other protein				
19	Aldose 1-epimerase	35.13	5.11	408
20/21	Catalase R	80.22	5.11	257
22	Hexokinase	54.46	5.13	71
24	GMC oxidoreductase	65.27	5.44	241
26	aminopeptidase C	72.67	5.56	74
27	Inorganic pyrophosphatase	37.07	5.65	312
28	spermidine synthase	33.73	5.42	262
30	Transaldolase	35.58	5.57	426
31	L-xylulose reductase	31.23	5.52	117
37	heat shock protein	65.40	5.34	603
38	thiJ/PfpI family protein	23.77	4.96	339
40	nitroreductase family protein	24.15	5.17	351
41/43	superoxide dismutase (Mn)	30.27	6.21	247/201
42	Aldo-keto reductases	36.95	5.97	116
44	NADPH-dependent FMN reductase	25.13	5.95	98
45/46	Peroxiredoxin pmp20	18.69	5.38	170
50	translation elongation factor 1	24.78	4.41	56
51	hypothetical protein	17.30	4.76	58

^1^Spot numbers correspond to the 2-DE gel in Fig 4B. The spots were detected in triplicate experiments.

^2^PI: isoelectric point

^3^ Proteins were successfully identified based on 95% or higher confidence interval of their scores in MASCOT V2.3 search engine (Matrix Science Ltd., London, U.K.)MASCOT score are derived from ions scores as a non-probabilistic basis for ranking protein hits. Moscot score greater than 53 was regarded as significant (*P* < 0.05).

## Discussion

Fermented rapeseed meal (FRSM) contained more CP and small peptide than unfermented RSM, which is consistent with previous experiment reports [25–26]. The loss of dry matter during fermentation may be a possible reason for the increase in protein [27]. Crude protein increased after fermentation, the amino acid contents of FRSM would also be increased except for His, which may be attributable to the preferential utilization of His by *A*. *niger*. Furthermore, FRSM also exhibited an increase in small peptide compared with untreated RSM. Uckun et al. 2012 showed that after 72 h of solid state fermentation with *Aspergillus oryzae*, free amino nitrogen production was increased to 34.5 mg/g which is equivalent to 55% conversion from the total protein in RSM [28]. An increased amount of small peptide in FRSM might be due to the digestion of large-size peptides in RSM by proteases secreted by *A*. *niger*. In this study, the AA contents (except His) of FRSM were increased compared to unfermented RSM, which is consistent with previous report [29]. In previous reports, Rakariyatham and Sakorn 2002 found that 100 μmol/g of glucosinolates was totally degraded by solid state fermentation with *Aspergillus sp*. NR-4201 in 48 h [30]. Chiang et al. 2010 reported that isothiocyanates were reduced from 119.6 to 14.7 mmol/kg in a 30 day composite strains fermentation [25]. In the present study, FRSM also exhibited a decline in glucosinolates and its degradation products (isothiocyanate and oxazolidithione) compared with control. Previous study found that protease and hemicellulase significantly decreased glucosinolates in rapeseed meal [31]. In this study, decrease of glucosinolates in rapseed meal might be due to many enzymes secreated by *A*. *niger* during SSF. The most substantially effect was the reduction in the phytic acid content in the RSM substrate which declined by about 86%. Reduction of phytic acid in present study is in agreement with findings of El-Batal and Karem 2001, who reported that *A*. *niger* had ability to secret phytase which causes the breakdown of phytate during the fermentation of RSM [32]. In fact, Phytase A secreated by *A*. *niger* had also been successfully identified in the present study.

In RSM, cellulose formed network-like structure and the other biopolymers (protein, fat and polysaccharides) were embedded in the core of this structure. Wang et al. 2012 compared the distribution of biopolymers in raw RSM and RSM after 14 days of composting. The results suggested the network-like structure of cellulose in the composted rapeseed meal was partly broken [20]. In the present study, SEM analysis showed that *A*. *niger* fermentation disrupted the surface structure of RSM substrate. The surface of the RSM substrate after SSF with A. *niger* was rough and irregular, which is beneficial in increasing the reactive surface area of RSM and improving enzymatic lysis reaction. Extracellular hydrolases (especially lignocellulolytic enzymes) secreted during SSF by *Aspergillus niger* could change the surface structure of RSM.

The FTIR spectroscopy technique can be applied to examine the structural changes in the biomass during pretreatments [33]. The FTIR bands that range between 3700 and 3100 cm^-1^ are usually used to investigate the OH bonds in cellulose [34]. In this study, some weak peaks at 3192, 3054 and 2969 cm^-1^, which is related to O-H stretching band of hydroxyl group, were observed in FTIR spectra of FRSM. This indicate chemical structure of cellulose in RSM might be changed during solid state fermentation. In addition, the absorbance at about 1620 cm^-1^, which is related to amide I group in proteins compounds (carbonyl stretching vibrations of the peptide backbone) [20], was increased after pretreatment by SSF with *A*. *niger*. The spectrum at 1080 cm^−1^ represents the C-O stretching of polysaccharides or polysaccharide-like substances [20]. The band intensity at this wave number for FRSM was significantly lower than that of the untreated RSM. According to Noda’s rule (2004), the degradation of biopolymers in rapeseed meal composts may follow the sequence: cellulose > heteropolysaccharides > amide II > amide I [35]. From the results above, solid state fermentation with *A*. *niger* may change the structure of cellulose and polysaccharides, while increased the ratio of amide group according to the change of absorption peak intensity.

Rapeseed protein contains two predominant classes: 12S globulin which represents 65% of its protein content and 2S albumin. The molecular mass of 12S globulin was estimated to be around 300 kDa. At extreme pH and in urea solutions, the globulin totally dissociates into six subunits, each of them being composed of two polypeptide chains (α and β) of about 30 and 20 kDa linked by a disulfide bond [36]. In our study, the band at 55 kDa was main rapeseed protein in raw RSM which is consistent with previous report. Solid state fermentation with *A*. *niger* affected the characteristics of proteins in RSM. Fermentation increased the amount of small-size peptides (< 35 kDa) compared with untreated RSM, while significantly decreasing large-size peptides (> 60 kDa). Hong et al. 2004 and Chen et al. 2010 also indicated that the large size protein fraction was significantly degraded during solid state fermentation of soybean meal [20, 37]. In our previous study, the enzyme activity of acid protease was also significantly increased during SSF of rapeseed meal [2]. Therefore, the increasing amounts of small size protein may be due to partial digestion of large size protein in RSM by protease secreted by *A*. *niger* during fermentation.

To further understand possible mechanism of solid state fermentation, the composition of extracellular enzymes secreted by *A*. *niger* during fermentation was analysed using 2D-DIGE combined with MALDI-TOF/TOF tandem MS. In the present study, the secretome profile of *A*. *niger* CICC41258 in FRSM was described for the first time. There were 40 successfully identified protein spots. In identified extracellular proteins, the majority of these proteins are hydrolytic enzymes involved in the degradation of cell wall polymers (beta-1,3/4-glucanase, arabinofuranosidase, endo-1,4-beta-xylanase), starch (glucoamylase), protein (aspergillopepsin A and protease B) and phytate (phytase A). In our previous report, several enzyme activities (endoglucanase, xylanase, acid protease and phytase) during SSF of RSM with *A*. *niger* were determined. We found that the activity of these enzymes also significantly increased with the fermentation prolonged [2]. Glucoamylase belongs to glycosyl hydrolase 15 family and hydrolyses terminal 1-4-linked alpha-D-glucose residues successively from nonreducing ends of the chains with release of beta-D-glucose. In this study, 4 protein spots were identified as starch hydrolytic related enzymes. This indicated that *A*. *niger* has strong ability to hydrolyze starch of RSM during SSF. Glucoamylase is the most efficiently secreted protein of *A*. *niger*, the glucoamylase (glaA) promoter as well as the signal sequence are widely used for heterologous protein production [38]. Aspergillopepsin, also known as aspartic proteinase, is a subfamily of endopeptidases that cleave peptide bonds within the polypeptide chain at acidic pH [39]. In our study, 3 protein spots (spot 5, 6 and 7) were identified as aspergillopepsin A and B, which indicated *A*. *niger* has ability to degrade marcomolecules peptides into small molecular weight peptides by proteases during fermentation. Phosphorus from plant-derived feedstuffs is mainly in the form of phytic P. In RSM, 80.5% of total P is bound in phytic acid and cannot be metabolized by animals [40]. Phytase is an enzyme that hydrolyses phytic acid to inositol and inorganic phosphorus, leading to improved phosphorus utilization and overall growth performance of monogastric animals [3]. In our study, 2 protein spots were identified as Phytase A. This is consistent with our result of chemical analysis. Other proteins found in the medium of *A*. *niger* grown on RSM are beta-1,3-glucanosyltransferase (Bgt1) and Carboxylesterase (Ces). Bgt1 is responsible for the elongation of 1,3-β-glucan chains during cell wall synthesis [41]. Ces from *A*. *niger* consisted of two identical subunits (each with a molecular weight of 60 kDa) catalyze the hydrolysis of ester bonds, where both the alcohol moiety and the acid moiety may be aliphatic as well as aromatic [42]. Moreover, several antioxidant related enzymes (Catalase R, Superoxide dismutase and Peroxiredoxin pmp20) which protects cells against oxidative damage [38], also were identified. The biodegradation of lignin is an oxidative process in which various oxidases and peroxidases enzymes might a major role [10]. Catalase and Peroxiredoxin suggested a partial lignin degrading potential of *A*. *niger*. Some intracellular proteins were also identified, suggesting cell damage during cell division or during sample processing. These intracellular proteins (mainly oxidoreductase) in the secretome of *A*. *niger* play an important role in protein and energy metabolism, which is essential for the maintenance of normal physiological function of the fungus during SSF.

These findings suggested that solid state fermentation with *A*.*niger* had significant effects on chemical composition of RSM by increasing small peptide and decreasing anti-nutritional substances. *A*. *niger* fermentation disrupted the surface structure, changed macromolecular organic compounds, and reduced protein molecular weight of RSM substrate. The secretome profile of *A*. *niger* in FRSM showed that *A*. *niger* is able to secrete many extracellular degradation enzymes (especially lignocellulosic hydrolyzing enzymes, acid proteases and phytase) during fermentation of RSM. These enzymes secreted by *A*. *niger* might result in the improvement of nutritional value of RSM.

## Conclusion

Solid state fermentation with *A*.*niger* could be practical methods for altering physicochemical properties of RSM. Many extracellular degradation enzymes (especially lignocellulosic hydrolyzing enzymes, acid proteases and phytase) secreted by *A*.*niger* might play important role in the improvement of nutritional value of RSM.

## Supporting Information

S1 Table Raw data of chemical composition of FRSM and RSM in Table 1. (XLSX)

## References

[1] Lomascolo A, Uzan BE, Sigoillot JC, Fine F. Rapeseed and sunflower meal: a review on biotechnology status and challenges. Appl Microbiol Biotechnol. 2012; 95: 1105–1114. 10.1007/s00253-012-4250-6 22752367

[2] Shi CY, He J, Yu J, Yu B, Huang ZQ, Mao XB et al *Aspergillus niger* for degrading glucosinolates and upgrading nutritional value. J Anim Sci Biotechno. 2015;6: 13. 10.1186/s40104-015-0015-2 PMC4399751 25883784

[3] Khajali F, Slominski BA. Factors that affect the nutritive value of canola meal for poultry. Poult Sci. 2012;91: 2564–2575. 22991543 10.3382/ps.2012-02332

[4] Tripathi MK, Mishra AS. Glucosinolates in animal nutrition: A review. Anim Feed Sci Tech. 2007;132: 1–27.

[5] Guggenbuhl P, Simões-Nunes C. Effects of two phytases on the ileal apparent digestibility of minerals and amino acids in ileo-rectal anastomosed pigs fed on a maize–rapeseed meal diet. Livest Sci. 2007;109: 261–263.

[6] Bau HM, Villaume C, Lin CF, Evrad J, Quemener B, Nicolas JP, et al Effect of a solid-state fermentation using *Rhizopus oligosporus* sp.T-3 on elimination of antinutritional substances and modification of biochemical constituents of defatted rapeseed meal. J Sci Food Agric. 1994;65: 315–322.

[7] Smit JP, Janssens RJJ, Knol P, Bol J. Modeling of the glucosinolate content in solid-state fermentation of rapeseed meal with fuzzy-Logic. J Ferment Bioeng. 1994;77: 579–581.

[8] Vig AP, Walia A. Beneficial effects of *Rhizopus oligosporus* fermentation on reduction of glucosinolates, fibre and phytic acid in rapeseed (*Brassica napus*) meal. Bioresour Technol. 2001;78: 309–312. 11341693 10.1016/s0960-8524(01)00030-x

[9] Wang XS, Jin QZ, Wang T, Huang JH, Xia YX, Yao LX, et al Screening of glucosinolate-degrading strains and its application in improving the quality of rapeseed meal. Ann Microbiol. 2012;62: 1013–1020.

[10] Adav SS, Li AA, Manavalan A, Punt P, Sze SK. Quantitative iTRAQ secretome analysis of *Aspergillus niger* reveals novel hydrolytic enzymes. J Proteome Res. 2010;9: 3932–3940. 10.1021/pr100148j 20545367

[11] Shi CY, He J, Yu J, Yu B, Mao XB, Zheng P, et al Amino acid, phosphorus, and energy digestibility of *Aspergillus niger* fermented rapeseed meal in growing pigs. J Anim Sci. 2015;93: 2916–2925. 10.2527/jas.2014-8326 26115278

[12] Oso AO, Li L, Zhang B, Uo R, Fan JX, Wang S, et al Effect of fungal fermentation with *Aspergillus niger* and enzyme supplementation on metabolisable energy values of unpeeled cassava root meal for meat-type cockerels. Anim Feed Sci Tech. 2015;210:281–286.

[13] Holst DO. Holst filtration apparatus for Van Soest detergent fiber analysis. J Assoc Off Anal Chem. 1973;56: 1352–1356.

[14] AOAC. Official methods of analysis of the association of official analytical chemistry. 18th edn. Assoc Anal Chem, Arlington, VA 2005.

[15] Rutherfurd SM. Methodology for determining degree of hydrolysis of proteins in hydrolysates: a review. J AOAC Int. 2010;93: 1515–1522. 21140664

[16] Ovissipour M, Abedian A, Motamedzadegan A, Rasco B, Safari R, Shahiri H. The effect of enzymatic hydrolysis time and temperature on the properties of protein hydrolysates from Persian sturgeon (*Acipenser persicus*) viscera. Food Chem. 2009;115: 238–242.

[17] Wathelet JP, Wagstaffe PJ, Biston R, Marlier M, Severin M. Rapeseed reference materials for glucosinolate analysis. Fresen J Anal Chem. 1988;332: 689–693.

[18] Choi MM, Shuang S, Lai HY, Cheng SC, Cheng RC, Cheung BK, et al Gas chromatography-mass spectrometric determination of total isothiocyanates in Chinese medicinal herbs. Analytica Chimica Acta. 2004;516: 155–163.

[19] Ellis R, Morris ER, Philpot C. Quantitative determination of phytate in the presence of high inorganic phosphate. Anal Biochem. 1977;77: 536–539. 842837 10.1016/0003-2697(77)90269-x

[20] Wang LP, Shen QR, Yu GH, Ran W, Xu YC. Fate of biopolymers during rapeseed meal and wheat bran composting as studied by two-dimensional correlation spectroscopy in combination with multiple fluorescence labeling techniques. Bioresour Technol. 2012;105: 88–94. 10.1016/j.biortech.2011.11.064 22182472

[21] Faurobert M. Application of two-dimensional gel electrophoresis to *Prunus armeniaca* leaf and bark tissues. Electrophoresis. 1997;17: 170–173. 10.1002/elps.1150180130 9059840

[22] Hong KJ, Lee CH, Kim SW. *Aspergillus oryzae* GB-107 fermentation improves nutritional quality of food soybeans and feed soybean meals. J Med Food. 2004;7: 430–436. 15671685 10.1089/jmf.2004.7.430

[23] Zhang B, Guan ZB, Cao Y, Xie GF, Lu J. Secretome of *Aspergillus oryzae* in Shaoxing rice wine koji. Int J Food Microbiol. 2012;155:113–119. 10.1016/j.ijfoodmicro.2012.01.014 22341915

[24] Pel HJ, de Winde JH, Archer DB, Dyer PS, Hofmann G, Schaap PJ, et al Genome sequencing and analysis of the versatile cell factory *Aspergillus niger* CBS 513.88. Nature Biotechnol. 2007;25: 221–231. 17259976 10.1038/nbt1282

[25] Chiang G, Lu WQ, Piao XS, Hu JK, Gong LM, Thacker PA. Effects of feeding solid-state fermented rapeseed meal on performance, nutrient digestibility, intestinal ecology and intestinal morphology of broiler chickens. Asian-Austral J Anim Sci. 2010;23: 263–271.

[26] Xu FZ, Li LM, Xu JP, Qian K, Zhang ZD, Liang ZY. Effects of fermented rapeseed meal on growth performance and serum parameters in ducks. Asian-Aust J Anim Sci. 2011;24: 678–684.

[27] Rozan P, Villaume C, Bau H, Schwertz A, Nicolas J, Mejean L. et al Detoxication of rapeseed meal by *Rhizopus Oligosporus sp*-T3: A first step towards rapeseed protein concentrate. Int J Food Sci Tech. 1996;31: 85–90.

[28] Uckun KE, Salakkam A, Trzcinski AP, Bakir U, Webb C. Enhancing the value of nitrogen from rapeseed meal for microbial oil production. Enzyme Microb Technol. 2012;50: 337–342. 10.1016/j.enzmictec.2012.03.004 22500902

[29] Khalaf MA, Meleigy SA. Reduction of free gossypol levels in cottonseed meal by microbial treatment. Int J Agric Biol. 2008;10: 185–190.

[30] Rakariyatham N, Sakorn P. Biodegradation of glucosinolates in brown mustard seed meal (brassica juncea) by *Aspergillus sp*. Nr-4201 in liquid and solid-state cultures. Biodegradation. 2002;13: 395–399. 12713131 10.1023/a:1022851129684

[31] Mahajan A, Dua S. Role of enzymatic treatments in modifying the functional properties of rapeseed (brassica campestris var.Toria) meal. Int J Food Sci Nutr. 1998;49: 435–440.

[32] El-Batal AI, Abdel KH. Phytase production and phytic acid reduction in rapeseed meal by *Aspergillus niger* during solid state fermentation. Food Res Int. 2001;34: 715–720.

[33] Oh SY, Yoo DI, Shin Y, Seo Get. FTIR analysis of cellulose treated with sodium hydroxide and carbon dioxide. Carbohydr Res. 2005;340: 417–428. 15680597 10.1016/j.carres.2004.11.027

[34] Hishikawa Y, Inoue S, Magoshi J, Kondo T. Novel tool for characterization of noncrystalline regions in cellulose: a FTIR deuteration monitoring and generalized two-dimensional correlation spectroscopy. Biomacromolecules. 2005;6: 2468–2473. 16153082 10.1021/bm050032k

[35] Noda I, Ozaki Y. Two-Dimensional Correlation Spectroscopy Applications in Vibrational and Optical Spectroscopy. Hoboken: John Wiley and Sons Press; 2004.

[36] Berot S, Compoint JP, Larre C, Malabat C, Guequen J. Large scale purification of rapeseed proteins (*Brassica napus*). J Chromatogr B. 2005;818: 35–42. 10.1016/j.jchromb.2004.08.001 15722042

[37] Chen CC, Shih YC, Chiou PWS, Yu B. Evaluating nutritional quality of single stage-and two stage-fermented soybean meal. Asian-Aust J Anim Sci. 2010;23: 598–606.

[38] Lu X, Sun JB, Nimtz M, Wissing J, Zeng AP, Rinas U. The intra- and extracellular proteome of *Aspergillus niger* growing on defined medium with xylose or maltose as carbon substrate. Microb Cell Fact. 2010;9: 23 10.1186/1475-2859-9-23 20406453 PMC2874515

[39] Hsiao NW, Chen Y, Kuan YC, Lee YC, Lee SK, Chan HH, et al Purification and characterization of an aspartic protease from *Rhizoupus oryzae* protease extract, Peptidase R. Electron J Biotechn. 2014;17: 89–94.

[40] Segueilha L, Lambrechts C, Boze H, Moulin G, Galzy P. Purification and properties of phytase from *Schwanniomyces castellii*. J Ferm Bioeng. 1992;74: 7–11.

[41] Mouyna I, Hartland RP, Fontaine T, Diaquin M, Simenel C, Delepierre M, et al A 1,3-beta-glucanosyltransferase isolated from the cell wall of *Aspergillus fumigatus* is a homologue of the yeast Bgl2p. Microbiology. 1998;144: 3171–3180. 9846753 10.1099/00221287-144-11-3171

[42] Li XM, Klaus BD. A novel carboxylesterase from *Aspergillus niger* and its hydrolysis of succinimide esters. Carlsberg Res Commun. 1989;54: 241–249.

